# Output Feedback Fractional-Order Nonsingular Terminal Sliding Mode Control of Underwater Remotely Operated Vehicles

**DOI:** 10.1155/2014/838019

**Published:** 2014-05-25

**Authors:** Yaoyao Wang, Jiawang Chen, Linyi Gu

**Affiliations:** ^1^State Key Lab of Fluid Power Transmission and Control, Zhejiang University, Hangzhou 310027, China; ^2^Ocean College, Zhejiang University, Hangzhou 310058, China

## Abstract

For the 4-DOF (degrees of freedom) trajectory tracking control problem of underwater remotely operated vehicles (ROVs) in the presence of model uncertainties and external disturbances, a novel output feedback fractional-order nonsingular terminal sliding mode control (FO-NTSMC) technique is introduced in light of the equivalent output injection sliding mode observer (SMO) and TSMC principle and fractional calculus technology. The equivalent output injection SMO is applied to reconstruct the full states in finite time. Meanwhile, the FO-NTSMC algorithm, based on a new proposed fractional-order switching manifold, is designed to stabilize the tracking error to equilibrium points in finite time. The corresponding stability analysis of the closed-loop system is presented using the fractional-order version of the Lyapunov stability theory. Comparative numerical simulation results are presented and analyzed to demonstrate the effectiveness of the proposed method. Finally, it is noteworthy that the proposed output feedback FO-NTSMC technique can be used to control a broad range of nonlinear second-order dynamical systems in finite time.

## 1. Introduction

Fractional calculus, an extension of ordinary integer-order differential and integral calculus, is a 300-year-old mathematical subject. However, its application to engineering and physics has only recently attracted significant attention [1–3]. One active field of the applications is fractional-order controller design.

After the concept of the fractional-order (FO) controller was first proposed [4], many FO control strategies combined with other classical control methods were proposed and investigated for both linear and nonlinear systems. One of these attractive control strategies is the combination of the FO control method and sliding mode control (SMC) technology [5, 6].

As one of the most significant achievements in modern control theory, SMC is a well-known efficient control scheme for handling control problems with model uncertainties and external disturbances [7]. Therefore, SMC has been widely applied in many systems such as underwater vehicles [8–13], mobile manipulator [14], stochastic Markovian jumping systems [15], near space vehicles [16], hysteretic structural systems [17], and chaotic systems [18, 19]. SMC mainly contains two components: a driving part that forces the system states to reach and stay on a stable predescribed sliding surface and a sliding surface that ensures the desired error dynamics of the systems [20]. Usually, the sliding surface is described by arbitrary linear dynamics, and it can only guarantee asymptotic stability, which means the system states need infinite time to converge to the equilibrium point. However, it is widely believed that the finite-time stabilization of dynamical systems may give rise to a high-precision and fast system dynamic response [21]. Thus, terminal sliding mode control (TSMC) and its improved version, nonsingular TSMC (NTSMC), which are variant schemes of traditional SMC and can achieve finite-time stability, were proposed and investigated [21–28]. Inspired by this idea, some control strategies combining fractional calculus and TSMC/NTSMC have been reported for both fractional-order and integer-order systems in the past few years [29–32]. It has been verified that the fractional-order TSMC/NTSMC method can ensure better control performance than the integer-order ones even for the integer-order plants.

Recalling the development of the fractional-order SMC/TSMC/NTSMC methods over the past few years, almost all theoretical studies and practical applications have focused on the full state feedback control strategies, which, however, may be unsuitable in many practical applications due to the immeasurability of the full states. Although a fractional-order dynamic output feedback sliding mode controller has been reported recently [33], it should be mentioned that the method proposed in [33] is designed for a class of fractional-order nonlinear systems and that the traditional linear-hyperplane-based SMC method was adopted instead of TSMC or NTSMC. Meanwhile, to the best of the authors' knowledge, there has been no study on the development of an output feedback fractional-order TSMC/NTSMC (FO-TSMC/FO-NTSMC) strategy for integer-order systems. Therefore, designing an output feedback fractional-order TSMC/NTSMC (FO-NTSMC/FO-NTSMC) strategy for the integer-order systems still remains an open and challenging problem to be solved.

Thus, in light of the equivalent output injection sliding mode observer (SMO) [34, 35], TSMC technology, and fractional calculus, we propose an output feedback FO-NTSMC scheme for underwater remotely operated vehicles (ROVs), a classical multivariable nonlinear second-order dynamic system, in this paper for the first time. The effects of model uncertainties and external disturbances are also taken into account and the proposed control scheme is able to tackle all of these uncertainties in the system dynamics. The main contributions of this paper are as follows: (1) design an equivalent output injection SMO for ROVs and present the corresponding proof; (2) design a novel fractional-order nonsingular terminal sliding manifold which is applicable for the classical second-order systems of ROVs; (3) design a novel control law to guarantee the reachability of the proposed sliding manifold; (4) prove the finite time stabilization of the closed-loop observer-controller systems with fractional-order dynamics for second-order systems. Finally, the goal of this control scheme is to control ROVs to track the desired trajectory in finite time using only the plants' output signal in the presence of model uncertainties and external disturbances.

The rest of this paper is organized as follows. In Section 2, some basic definitions and preliminaries of fractional-order calculus are presented. In Section 3, the system description and problem formulation are introduced. In Section 4, the integer-order TSMC and NTSMC are briefly reviewed. In Section 5, the design procedure of the proposed fractional-order output feedback FO-NTSMC is demonstrated. Corresponding stability and reachability analyses are performed. In Section 6, the validation of the proposed method is verified through numerical simulations. Finally, some conclusions are presented in Section 7.

## 2. Preliminaries

In this section, basic definitions of fractional calculus and a necessary fractional calculus stability lemma are presented. Two of the most commonly adopted definitions are the Riemann-Liouville and Caputo definitions.

**Definition 1 (see [36])**

The *α*th-order Riemann-Liouville fractional derivative of function *f*(*t*) with respect to *t* and the terminal value *t*_0_ is defined as

(1) $$\begin{array}{c} D^{\alpha }f\left(t\right)=\frac{{\text{d}}^{\alpha }f\left(t\right)}{\text{d}t^{\alpha }}=\frac{1}{\Gamma \left(m-\alpha \right)}\frac{{\text{d}}^{m}}{\text{d}t^{m}}\overset{t}{\underset{t_{0}}{\int }}\frac{f\left(\tau \right)}{{\left(t-\tau \right)}^{\alpha -m+1}}\text{d}\tau \end{array}$$

and the *α*th-order Riemann-Liouville fractional integration is defined as

(2) Itαt0f(t)=1Γ(α)∫t0tf(τ)dτ(t−τ)1−α,

where *m* − 1 < *α* ≤ *m*, *m* ∈ *N*, and Γ(·) is the Gamma function.

**Definition 2 (see [36])**

The Caputo fractional derivative of order *α* of a continuous function *f*(*t*) is defined as follows:

(3) $$\begin{array}{c} D^{\alpha }f\left(t\right)=\{\begin{array}{cc} \frac{1}{\Gamma \left(m-\alpha \right)}\overset{t}{\underset{0}{\int }}\frac{f^{\left(m\right)}\left(\tau \right)}{{\left(t-\tau \right)}^{\alpha -m+1}}\text{d}\tau , & m-1<\alpha <m\\ \frac{{\text{d}}^{m}}{\text{d}t^{m}}f\left(t\right), & \alpha =m, \end{array} \end{array}$$

where *m* is the first integer larger than *α*.

**Property 1 (see [36])**

If the fractional derivative _*t*_0__ *D*_*t*_^*α*^ *y*(*t*) (*k* − 1 ≤ *α* < *k*) of a function *y*(*t*) is integrable, then

(4) Itαt0(Dtαt0y(t))=y(t)−∑j=1k[Dtα−jt0y(t)]t=t0(t−t0)α−jΓ(α−j+1).

**Lemma 3 (see [37])**

The fractional integration operator _*t*_0__ *I*_*t*_^*α*^ with ⌊*α*⌋ > 0 is bounded as

(5) $$\begin{array}{c} {||I^{\alpha }y||}_{p}\leq K{||y||}_{p},\;1\leq p\leq \infty . \end{array}$$

## 3. System Description and Problem Formulation

**Figure 1:**
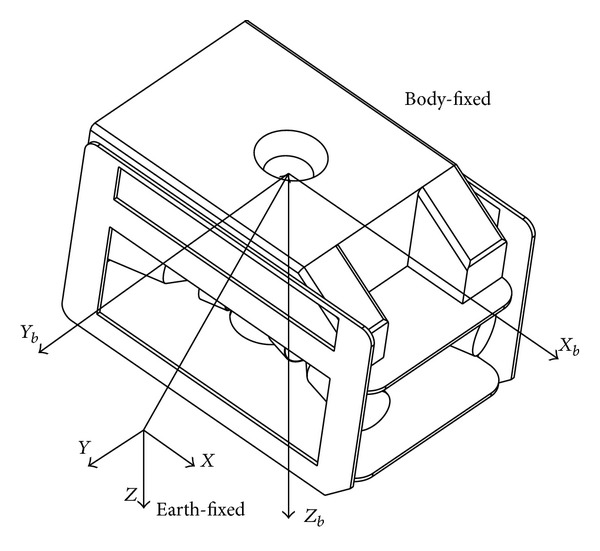
Earth-fixed and body-fixed frame.

The standard form of the kinematics and dynamics equations of ROVs in 4-DOF, described in the earth-fixed coordinate and body-fixed coordinate frames as indicated in Figure 1, can be written as follows [38]:

(6) $$\begin{array}{c} \dot{\eta }=J\left(\eta \right)v,\\ M\dot{v}+C\left(v\right)v+D\left(v\right)v+g\left(\eta \right)=\tau +J^{T}\left(\eta \right)d, \end{array}$$

where *η* = [*x*, *y*, *z*, *ψ*]^*T*^ denotes the ROV's location and orientation in the earth-fixed coordinate, whereas *v* = [*u*, *v*, *w*, *r*]^*T*^ denotes the vector of the ROV's linear and angular velocity expressed in the body-fixed coordinate. *M* = *M*_0_ + Δ*M* ∈ *R* ^4×4^ is the inertial matrix including added mass. *C*(*v*) = *C*_0_(*v*) + Δ*C*(*v*) ∈ *R* ^4×4^ represents the Coriolis and centripetal forces. *D*(*v*) = *D*_0_(*v*) + Δ*D*(*v*) ∈ *R* ^4×4^ is the hydrodynamic damping term, and the vector *g*(*η*) = *g*_0_(*η*) + Δ*g*(*η*) ∈ *R* ^4×1^ is a combined force/moment of gravity and buoyancy in the body-fixed coordinate. *M*_0_, *C*_0_(*v*), *D*_0_(*v*), and *g*_0_(*η*) are the nominal parameter matrices, whereas Δ*M*, Δ*C*(*v*), Δ*D*(*v*), and Δ*g*(*η*) are the model uncertainties. *J* ^*T*^(*η*)*d* ∈ *R* ^4×1^ is the disturbance force/moment vector expressed in the body-fixed coordinate and *τ* ∈ *R* ^4×1^ is the system control input.

*J*(*η*) is the kinematic transformation matrix which expresses the transformation from the body-fixed frame to earth-fixed frame and can be expressed as follows:

(7) $$\begin{array}{c} J\left(\eta \right)=\left[\begin{array}{cccc} \cos\psi & -\sin\psi & 0 & 0\\ \sin\psi & \cos\psi & 0 & 0\\ 0 & 0 & 1 & 0\\ 0 & 0 & 0 & 1 \end{array}\right]. \end{array}$$

The other simplified parameter matrices can be expressed as follows:

(8) M0 =diag⁡{m−Xu˙,m−Yv˙,m−Zw˙,Iz−Nr˙},C0(v) =[000−(mv−Yv˙)v000−(mv−Xu˙)u0000(mv−Yv˙)v−(mv−Xu˙)u00],D0(v)=diag⁡{Xu+Xu|u|u,Yv+Yv|v|v,Zw+Zw|w|w,Nr+Nr|r|r},g0(η) =[0,0,W−B,0]T,

where *W* and *B* denote the weight and buoyancy of the ROV, respectively.

Before we present the main results, necessary preliminary information is provided [21, 34].

**Assumption 4 (see [34])**

The MIMO dynamic system given by (6) does not have a finite escape time.

**Assumption 5 (see [34])**

The control input *τ* belongs to the extended *L*_*p*_ space, denoted by *L*_*p*_′ in this paper. Any truncation of *τ* to a finite time interval is bounded.

**Assumption 6 (see [34])**

The desired trajectory *η*_*d*_ is smooth; that is, ${\dot{\eta }}_{d}$ and ${\ddot{\eta }}_{d}$ are bounded, exist, and are known.

**Lemma 7 (see [21])**

An extended Lyapunov description of finite-time stability can be given with form of fast TSM as

(9) V˙(x)+αV(x)+βVγ(x)≤0, α>0,  β>0,0<γ<1,

and the settling time can be given by

(10) $$\begin{array}{c} T\leq \frac{1}{\alpha \left(1-\gamma \right)}\ln\frac{\alpha V^{1-\gamma }\left(x_{0}\right)+\beta }{\beta }. \end{array}$$

## 4. Review of the Integer-Order TSM and NTSM

In this section, definitions of the TSM and NTSM are briefly introduced as a necessary preparation for the output feedback FO-NTSMC design.

**Definition 8 (see [21, 24])**

The TSM and NTSM are equivalent and can be, respectively, described by the following first-order nonlinear differential equations:

(11) $$\begin{array}{c} s=\dot{e}+\beta \;\text{sig}{\left(e\right)}^{\mu }=0,\\ s^{\prime }=e+\beta^{\prime }\text{sig}{\left(\dot{e}\right)}^{\mu^{\prime }}=0, \end{array}$$

where

(12) $$\begin{array}{c} \beta^{\prime }=\beta^{-1/\mu }=\beta^{-\mu^{\prime }}>0,\;1<\mu^{\prime }=\frac{1}{\mu }<2. \end{array}$$

The TSM and NTSM defined in (11) are continuous and differentiable despite the adoption of the absolute value and the signum operator; the first derivatives thereof can be, respectively, expressed as follows [21]:

(13) $$\begin{array}{c} s=\ddot{e}+\beta \mu {\left|e\right|}^{\mu -1}\dot{e},\\ s^{\prime }=\dot{e}+\beta^{\prime }\mu^{\prime }{\left|\dot{e}\right|}^{\mu^{\prime }-1}\ddot{e}. \end{array}$$

## 5. Main Results

In this section, we will develop an output feedback FO-NTSMC approach for the trajectory tracking control of ROVs in the presence of model uncertainties and external disturbances. First, an equivalent output injection SMO will be established to estimate the ROV's velocity. Then, a novel fractional-order nonsingular terminal sliding manifold will be proposed to ensure the desired dynamics. Finally, a control law is designed to force the trajectory to reach the designed sliding manifold in finite time and remain on it forever.

### 5.1. Equivalent Output Injection Sliding Mode Observer Design

In this subsection, the equivalent output injection SMO will be designed and analyzed. The following notation will be used except stated otherwise: $\hat{x}$ represents the estimation of *x* and *x*_*i*_ represents the *i*th component of the vector *x*. The math operations used between two vectors are performed in terms of the corresponding elements. And in this paper, *i* = 1 ~ 4.

The nonlinear model of ROVs in the earth-fixed coordinate is adopted here to simplify the SMO design procedure [38]:

(14) $$\begin{array}{c} \dot{\eta }=v_{e}=J\left(\eta \right)v, \end{array}$$

(15) M0′(η)v˙e+C0′(v,η)ve+D0′(v,η)ve+g0′(η) =J−T(η)τ+d,

where *v*_*e*_ is the velocity vector in the earth-fixed frame, and the parameter matrices in (15) can be described as follows:

(16) M0′(η)= J−T(η)M0J−1(η),C0′(v,η)= J−T(η)[C0(v)−M0J−1(η)J˙(η)]J−1(η),D0′(v,η)= J−T(η)D0(v)J−1(η),g0′(η)= J−T(η)g0(η).

*Property 2* (see [38]). The parameter matrices have some great properties in earth-fixed frame when *M*_0_ = *M*_0_^*T*^ and ${\dot{M}}_{0}=0$. Consider

(17) M0′(η)=M0′T(η)>0, ∀η∈R4×1,xT[M0′˙(η)−2C0′(v,η)]x=0, ∀x∈R4×1,v∈R4×1, η∈R4×1,D0′(v,η)>0, ∀v∈R4×1,  η∈R4×1.

Define *x*_1_ = *η* and *x*_2_ = *v*_*e*_. Then, according to (14) and (15), the following model of ROVs in the earth-fixed coordinate can be obtained:

(18) x˙1=x2,M0′(x1)x˙2=−C0′(x1,x2)x2−D0′(x1,x2)x2−g0′(x1)+J−T(η)τ+τd,

where $\tau_{d}=d-\Delta M^{\prime }{\dot{x}}_{2}-\Delta C^{\prime }x_{2}-\Delta D^{\prime }x_{2}-\Delta g^{\prime }\in R^{4\times 1}$ is the lumped uncertainty including model uncertainties and external disturbances.

**Assumption 9 (see [39])**

The lumped uncertainty *τ*_*d*_ is local Lipschitz continuous and can be bounded with a constant unknown vector *F*(·):

(19) $$\begin{array}{c} \left|\tau_{d}\right|<F\left(\cdot \right)\in R^{4\times 1}. \end{array}$$

**Remark 10**

In practical applications, the control inputs of ROVs are obviously bounded which means that if the lumped uncertainty *τ*_*d*_ is unbounded, we cannot effectively control the trajectory of the ROVs.

Inspired by [34, 39], the equivalent output injection SMO for ROVs is designed as follows:

(20) x^˙1=x^2+γ1sgn⁡(x~1),M0′(x1)x^˙2=−C0′(x1,x^2)x^2−D0′(x1,x^2)x^2−g0′(x1)+J−T(η)τ+γ2sgn⁡(x−2−x^2),

where *γ*_1_ ∈ *R* ^4×1^ and *γ*_2_ ∈ *R* ^4×1^ are positive constant vectors to be designed and ${\tilde{x}}_{1}=x_{1}-{\hat{x}}_{1}$ and ${\tilde{x}}_{2}=x_{2}-{\hat{x}}_{2}$ are estimation errors. Consider ${\overset{-}{x}}_{2}={\hat{x}}_{2}+{\left(\gamma_{1}\mathrm{sgn}({\tilde{x}}_{1})\right)}_{\text{eq}}\in R^{4\times 1}$; ${\left(\gamma_{1}\mathrm{sgn}({\tilde{x}}_{1})\right)}_{\text{eq}}$ is the equivalent output injection, which can be obtained by passing the signal $\gamma_{1}\mathrm{sgn}({\tilde{x}}_{1})\;$through a low pass filter; more details can be found in [35].

Thus, the observer error dynamics can be obtained in terms of (18) and (20):

(21) $$\begin{array}{c} {\dot{\tilde{x}}}_{1}={\tilde{x}}_{2}-\gamma_{1}\mathrm{sgn}\left({\tilde{x}}_{1}\right), \end{array}$$

(22) $$\begin{array}{c} M_{0}^{\prime }{\dot{\tilde{x}}}_{2}=-\gamma_{2}\mathrm{sgn}\left({\overset{-}{x}}_{2}-{\hat{x}}_{2}\right)+\tau_{d}+f\left(\cdot \right), \end{array}$$

where $f(\cdot )=C_{0}^{\prime }(x_{1},{\hat{x}}_{2}){\hat{x}}_{2}-C_{0}^{\prime }(x_{1},x_{2})x_{2}+D_{0}^{\prime }(x_{1},{\hat{x}}_{2}){\hat{x}}_{2}-D_{0}^{\prime }(x_{1},x_{2})x_{2}$.

**Theorem 11**

Under Assumptions 4–9, the equivalent output injection SMO (20) for ROVs can guarantee that the estimation errors ${\tilde{x}}_{1}$ and ${\tilde{x}}_{2}$ converge to 0 in finite time.

**Proof**

The proof procedure is similar to those presented in [34, 39] with the exception that a different dynamic model is used here. As demonstrated in [10], the sliding mode technology allows systems to be separately designed and analyzed for each DOF. Hence, the proof procedure will be presented in each separate DOF for simplicity. The proof will be presented in two steps.

*Step 1*. This step will prove that the estimation error ${\tilde{x}}_{1}$ will converge to zero in finite time. Choose a Lyapunov candidate for the estimation error dynamic (21) as

(23) $$\begin{array}{c} V_{1i}=\frac{1}{2}{{\tilde{x}}^{2}}_{1i}, \end{array}$$

where *i* = 1 ~ 4.

Differentiating *V*_1*i*_ with respect to time along (21) yields

(24) $$\begin{array}{c} {\dot{V}}_{1i}={\tilde{x}}_{1i}{\dot{\tilde{x}}}_{1i}=-\left|{\tilde{x}}_{1i}\right|\gamma_{1i}+{\tilde{x}}_{1i}{\tilde{x}}_{2i}\leq -\left|{\tilde{x}}_{1i}\right|\left[\gamma_{1i}-\left|{\tilde{x}}_{2i}\right|\right]. \end{array}$$

According to Assumptions 4, 5, and 9, the estimation error ${\tilde{x}}_{2i}$ does not have finite escape time. This effectively ensures that the estimation error ${\tilde{x}}_{2i}$ is in the *L*_*p*_′ space. Thus, if we choose $\gamma_{1i}>\left|{\tilde{x}}_{2i}\right|+\varepsilon_{1i}$, *ε*_1*i*_ > 0, then the following inequality can be obtained:

(25) $$\begin{array}{c} {\dot{V}}_{1i}\leq -\varepsilon_{1i}\left|{\tilde{x}}_{1i}\right|. \end{array}$$

Therefore, the finite time convergence of ${\tilde{x}}_{1i}$ to 0 will be guaranteed. Choose $\gamma_{1i}>\max_{t\in [0,T^{\prime }]}\left|{\tilde{x}}_{2i}\right|$, where *T*′ is chosen large enough that $\gamma_{1i}>\left|{\tilde{x}}_{2i}\right|+\varepsilon_{1i}$; thus inequality (25) will always hold. Taking the fact that $\left|{\tilde{x}}_{1i}\right|=\sqrt{2}V_{1i}^{1/2}$ into consideration, we have

(26) $$\begin{array}{c} {\dot{V}}_{1i}=-\sqrt{2}\varepsilon_{1i}V_{1i}^{1/2}. \end{array}$$

Using the differential inequality principle [40, 41], we can conclude that *V*_1*i*_ = 0 when $t_{1i}\geq t_{0i}+\left(\left|{\tilde{x}}_{1i}(t_{0i})\right|/\varepsilon_{1i}\right)$, where *t*_0*i*_ is the initial time. Furthermore, when *t*_*i*_ > *t*_1*i*_, ${\tilde{x}}_{1i}=0$. Hence, on the sliding mode, ${\tilde{x}}_{1i}={\dot{\tilde{x}}}_{1i}=0$ and ${\tilde{x}}_{2}={\left(\gamma_{1}\mathrm{sgn}({\tilde{x}}_{1})\right)}_{\text{eq}}$. Thus, we have $\mathrm{sgn}({\overset{-}{x}}_{2i}-{\hat{x}}_{2i})=\mathrm{sgn}{\left((\gamma_{1i}\mathrm{sgn}({\tilde{x}}_{1i})\right)}_{\text{eq}})=\mathrm{sgn}({\tilde{x}}_{2i})$. Therefore, the observer error dynamics (21)-(22) of the *i*th component can be rearranged as follows:

(27) $$\begin{array}{c} {\dot{\tilde{x}}}_{1i}=0,\\ M_{0i}^{\prime }{\dot{\tilde{x}}}_{2i}=-\gamma_{2i}\mathrm{sgn}\left({\tilde{x}}_{2i}\right)+\tau_{di}+f{\left(\cdot \right)}_{i}. \end{array}$$

*Step 2*. We will prove that the estimation error ${\tilde{x}}_{2i}$ will converge to zero in finite time. Choose Lyapunov function candidate *V*_2*i*_ as follows:

(28) $$\begin{array}{c} V_{2i}=\frac{1}{2}{{\tilde{x}}^{2}}_{1i}+\frac{1}{2}M_{0i}^{\prime }{{\tilde{x}}^{2}}_{2i}. \end{array}$$

Since ${\tilde{x}}_{1i}=0$ when *t*_*i*_ > *t*_1*i*_, differentiating *V*_2*i*_ along (27) yields

(29) V˙2i=(−γ2sgn⁡(x~2)+τd+f(·))ix~2i=−γ2i|x~2i|+(τd+f(·))ix~2i≤−|x~2i|(γ2i−|(τd+f(·))i|).

If we choose *γ*_2__*i*_ ≥ |(*τ*_*d*_+*f*(·))_*i*_| + *ε*_2*i*_, *ε*_2*i*_ > 0 is a positive constant to be designed; then (29) can be rewritten as ${\dot{V}}_{2i}\leq -\varepsilon_{2i}\left|{\tilde{x}}_{2i}\right|$. Applying the same proof procedure indicated in Step 1, we can have that ${\tilde{x}}_{2i}$ will convergence to 0 as *t*_2*i*_ ≥ *t*_1*i*_ + (|*x*_2*i*_(*t*_1*i*_)|/*ε*_2*i*_). Therefore, the estimation errors ${\tilde{x}}_{1i}$ and ${\tilde{x}}_{2i}$ will converge to 0 in finite time. The proof is completed.

### 5.2. Output Feedback FO-NTSMC Design

In this subsection, a novel output feedback FO-NTSMC method for the 4-DOF trajectory tracking control of ROVs will be proposed and analyzed using the proposed equivalent output injection SMO. The design procedure mainly involves two steps. First, a novel nonlinear fractional-order nonsingular terminal sliding manifold will be proposed. Then, a control law will be designed to ensure the finite-time reachability of the proposed sliding manifold.

To simplify the application of the equivalent output injection SMO, the dynamic equations (20) will be rewritten in the body-fixed coordinate as follows:

(30) η^˙=J(η)v^+γ1sgn⁡(η~),M0v^˙=JT(η)γ2sgn⁡(J(η)v−−J(η)v^)−C0(v^)v^−D0(v^)v^−g0(η)+τ,

where $\tilde{\eta }=\eta -\hat{\eta }$ is the estimation error and $J(\eta )\overset{-}{v}=J(\eta )\hat{v}+{\left(\gamma_{1}\mathrm{sgn}(\tilde{\eta })\right)}_{\text{eq}}$. ${\left(\gamma_{1}\mathrm{sgn}(\tilde{\eta })\right)}_{\text{eq}}$ is the equivalent output injection of $\gamma_{1}\mathrm{sgn}(\tilde{\eta })$, which can be acquired by passing signal $\gamma_{1}\mathrm{sgn}(\tilde{\eta })$ through a low pass filter [34, 39].

Define the estimated tracking error and its derivative as

(31) e^1= η^−ηd,e^2= J(η)v^−η˙d.

Then, the estimated tracking error dynamic can be obtained as

(32) e^˙1 =e^2+γ1sgn⁡(η~),e^˙2=J(η)M0−1(JT(η)γ2sgn⁡(v→)−H0(v^,η)+τ)+J˙(η)v^−η¨d,

where $\vec{v}=J(\eta )\overset{-}{v}-J(\eta )\hat{v}$ and $H_{0}(\hat{v},\eta )=C_{0}(\hat{v})\hat{v}+D_{0}(\hat{v})\hat{v}+g_{0}(\eta )$.

In light of the TSM defined in (11), the novel fractional-order nonsingular terminal sliding mode (FO-NTSM) is designed as

(33) $$\begin{array}{c} \hat{s}={\hat{e}}_{2}+D^{\alpha -1}\left[\beta \;\text{sig}{\left({\hat{e}}_{1}\right)}^{\mu }\right], \end{array}$$

where 0 < *α* < 1, *β* > 0, and 1/2 < *μ* < 1 are positive parameter matrices to be designed.

A fast-TSM-type reaching law is adopted here [21]:

(34) $$\begin{array}{c} \dot{\hat{s}}=-k_{1}\hat{s}-k_{2}\text{sig}{\left(\hat{s}\right)}^{\rho }, \end{array}$$

where *k*_1_ > 0, *k*_2_ > 0, and 0 < *ρ* < 1 are positive parameter matrices to be designed.

Then, the output feedback FO-NTSMC is designed as follows:

(35) τ=τ1+τ2+τ3+τ4,τ1=H0(v^,η)+M0J−1(η)(η¨d−J˙(η)v^),τ2=−M0J−1(η)(Dα[β sig(e^1)μ]),τ3=−M0J−1(η)(k1s^+k2sig(s^)ρ),τ4=−M0J−1(η)Ksgn⁡(s^),

where *K* is a positive constant vector to be designed.

**Theorem 12**

Consider an estimated tracking error dynamic (32) subjected to the output feedback FO-NTSMC (35). Then, the estimated tracking errors ${\hat{e}}_{1}$ and ${\hat{e}}_{2}$ will converge to zero in finite time. Moreover, according to the principle of equivalent output injection SMO, the system trajectory tracking errors *e*_1_ and *e*_2_ will converge to 0 in finite time.

**Proof**

As demonstrated in [42], it is more appropriate to prove the occurrence of the sliding mode via fractional-order Lyapunov stability theorems [2, 43] when the closed-loop systems involve fractional-order dynamics.

Inspired by the proof procedure presented in [31], a Lyapunov function is selected as follows:

(36) $$\begin{array}{c} V_{3i}=\left|{\hat{s}}_{i}\right|. \end{array}$$

Then, differentiating (36) with respect to time yields

(37) $$\begin{array}{c} {\dot{V}}_{3i}={\dot{\hat{s}}}_{i}\mathrm{sgn}\left({\hat{s}}_{i}\right)={\left\{{\dot{\hat{e}}}_{2}+D^{\alpha }\left[\beta \;\text{sig}{\left({\hat{e}}_{1}\right)}^{\mu }\right]\right\}}_{i}\mathrm{sgn}\left({\hat{s}}_{i}\right). \end{array}$$

Substitute the estimated tracking error dynamic (32) and the control law (35) yields

(38) V˙3i=−{k1i|s^i|+k2i|s^i|ρi+Ki−(ξ(·)γ2sgn⁡(v→))isgn⁡(s^i)}≤−{k1|s^|+k2|s^|ρ}i−(Ki−γ2i||ξ(·)||),

where *ξ*(·) = *J*(*η*)*M*_0_^−1^ *J* ^*T*^(*η*).

If we choose *K*_*i*_ large enough such that *K*_*i*_ − *γ*_2*i*_||*ξ*(·)|| > 0, then inequality (38) can be rewritten as

(39) $$\begin{array}{c} {\dot{V}}_{3i}\leq -{\left\{k_{1}\left|\hat{s}\right|+k_{2}{\left|\hat{s}\right|}^{\rho }\right\}}_{i}=-k_{1i}V_{3i}-k_{2i}V_{3i}^{\rho_{i}}. \end{array}$$

According to Lemma 7, the finite time occurrence of the sliding mode can be guaranteed. And the settling time can be estimated as

(40) t3i≤1k1i(1−ρi)ln⁡k1iV1−ρi(s^(t0))+k2ik2i=1k1i(1−ρi)ln⁡k1i|s^(t0)|1−ρi+k2ik2i,

where $\hat{s}(t_{0})$ is the initial value of $\hat{s}(t)$.

The trajectory on the sliding manifold will be analyzed as follows. On the FO-NTSM, the behavior of the closed-loop system is dominated by the equivalent control law [7]. Differentiating the FO-NTSM with respect to time yields

(41) s^˙i={e^˙2+Dα[β sig(e^1)μ]}i={J(η)M0−1(JT(η)γ2sgn⁡(v→)−H0(v^,η)+τ) +J˙(η)v^−η¨d+Dα[β sig(e^1)μ]}i.

Then, the equivalent control law can be obtained as

(42) τeq=H0(v^,η)−JT(η)γ2sgn⁡(v→)+M0J−1(η)×(η¨d−J˙(η)v^−Dα[β sig(e^1)μ]).

When the trajectory is on FO-NTSM, we have $\hat{s}=0$. Furthermore, it is noteworthy that, according to Assumptions 4–9, the desired trajectory is bounded and smooth, and the estimated tracking errors ${\hat{e}}_{1}$ and ${\hat{e}}_{2}$ are in the set *L*_*p*_′. Thus, any finite truncation of the tracking error subject to the equivalent control will be bounded. In addition, according to Theorem 11, if the gains *γ*_1_ and *γ*_2_ are chosen appropriately, the estimated system states will converge to the real ones in finite time *t*_2*i*_ regardless of the stability of the closed-loop system. After $\overset{-}{t}=\max\left\{t_{2},t_{3}\right\}$, the estimated system states will converge to the real ones and stay on the FO-NTSM. Thus, during the FO-NTSM, substituting the equivalent control law (42) into the estimated tracking error dynamics (32) yields

(43) e˙1 =e2,e˙2 =−Dα[β sig(e1)μ],

where *e*_1_ = *η* − *η*_*d*_ and $e_{2}=J(\eta )v-{\dot{\eta }}_{d}$ are the real tracking errors of the closed-loop system.

Equation (43) can be rearranged as follows:

(44) $$\begin{array}{c} {\ddot{e}}_{1}=-D^{\alpha }\left[\beta \;\text{sig}{\left(e_{1}\right)}^{\mu }\right]. \end{array}$$

Now, we will prove that the tracking errors *e*_1_ and *e*_2_ will converge to zero in finite time using a proof procedure similar to that of [32]. Define a stopping time as follows:

(45) $$\begin{array}{c} t_{s}=\inf\left\{t\geq \overset{-}{t}:e\left(t\right)=0\right\}. \end{array}$$

According to Definition 1 and operator Dt-2t- and the associativity law, (44) can be rewritten as

(46) e1(t)−[Dtt−e1(t)]t=t−(t−t−)2−e1(t−) =Dtα−2t−[β sig(e1(t))μ].

According to Lemma 3, we have

(47) Dtα−2t−[β sig(e1(t))μ] =It2−αt−[β sig(e1(t))μ]≤Kβ||e1μ||.

Then, substituting (47) into (46) yields

(48) e1(t)−[Dtt−e1(t)]t=t−(t−t−)2−e1(t−)≤Kβ||e1μ||.

Equation (48) can be rearranged as

(49) ||e1(t)−[Dtt−e1(t)]t=t−(t−t−)2||−||e1(t−)||≤Kβ||e1μ||.

Noting that *e*_1_(*t*) = 0 at *t* = *t*_*s*_, it yields

(50) ||[Dtt−e1(t)]t=t−2||(ts−t−)≤||e1(t−)||.

If [Dtt-e1(t)]t=t-=0, then $t_{s}=\overset{-}{t}$. Otherwise, we have

(51) $$\begin{array}{c} t_{s}\leq \frac{2||e_{1}\left(\overset{-}{t}\right)||}{||{\dot{e}}_{1}\left(\overset{-}{t}\right)||}+\overset{-}{t}. \end{array}$$

Therefore, ROVs can track the desired trajectory in finite time using only the systems' output position signal. The proof is completed.

## 6. Simulation Results

In this section, some numerical simulations are performed to illustrate the effectiveness of the proposed method. The nominal physical parameters of the ROV are listed in Table 1.

The control parameters are as follows: *γ*_1*i*_ = 1.5, *γ*_2*i*_ = 80, *β*_*i*_ = 0.1, *μ*_*i*_ = 0.7, *k*_1*i*_ = 0.5, *k*_2*i*_ = 0.25, *ρ*_*i*_ = 0.9, *α*_*i*_ = 0.9, *K* = diag⁡{0.5, 0.5, 2, 0.5}, *η*(*t*_0_)_*i*_ = −0.2, *v*(*t*_0_)_*i*_ = 0, $\hat{\eta }{\left(t_{0}\right)}_{i}=0$, and $\hat{v}{\left(t_{0}\right)}_{i}=-0.05$. *η*(*t*_0_), *v*(*t*_0_), $\hat{\eta }(t_{0})$, and $\hat{v}(t_{0})$ are the initial values of the real and estimated position and velocity information. The desired trajectory is *η*_*di*_ = 0.3sin(0.05*πt*). To illustrate the robustness of the proposed method, time-varying disturbances *d*_*i*_ = 10sin(0.1*πt*) and a parametric variant of 20%, which indicate that the nominal physical parameters used in the output feedback FO-NTSMC are 20% less than those used in the model, are introduced into the closed-loop system. Furthermore, the dynamics of the propellers are also taken into account. We treat the propellers as one-order initial systems with a time constant of 0.5 seconds.

To compare with the integer-order control method expressed as output feedback IO-NTSMC, we adopt the NTSM manifold defined in the second equation of (11), and then the control law (35) will be changed to

(52) τ=τ1+τ2+τ3+τ4,τ1=H0(v^,η)+M0J−1(η)(η¨d−J˙(η)v^),τ2=−M0J−1(η)sig(e^2)2−μ′β′μ′,τ3=−M0J−1(η)(k1s^+k2sig(s^)ρ),τ4=−M0J−1(η)Ksgn⁡(s^),

where the new parameter matrices *β*′ and *μ*′ can be obtained using (12). The other parameters remain unchanged for the fairness of comparision.

**Figure 2:**
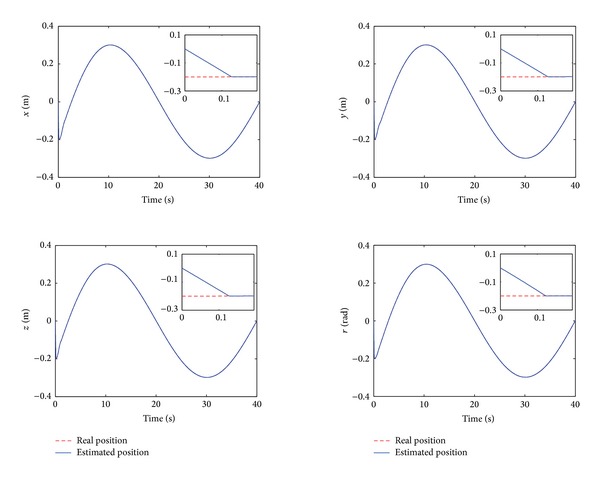
Estimated position and real position of output feedback FO-NTSMC.

**Figure 3:**
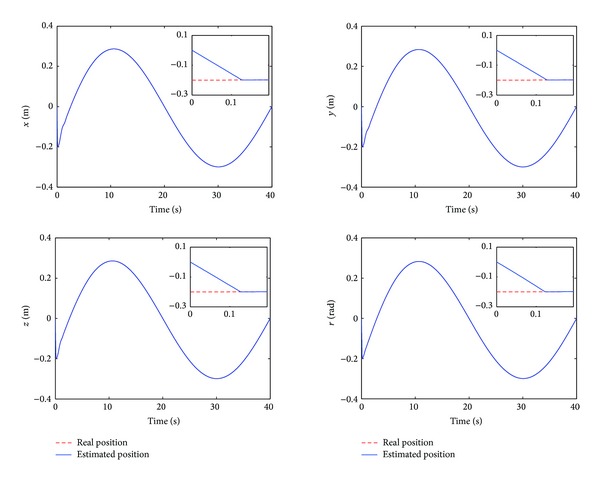
Estimated position and real position of output feedback IO-NTSMC.

**Figure 4:**
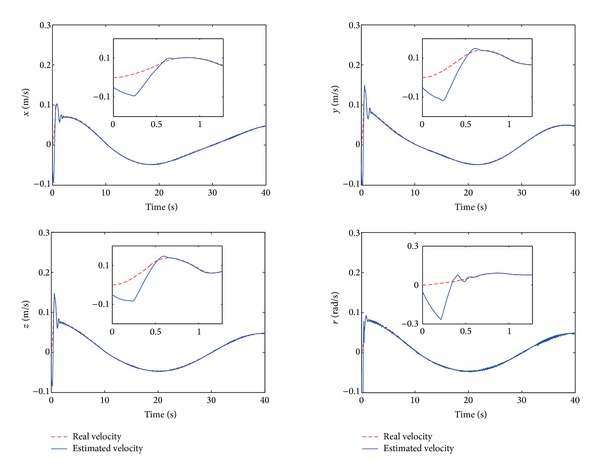
Estimated velocity and real velocity of output feedback FO-NTSMC.

**Figure 5:**
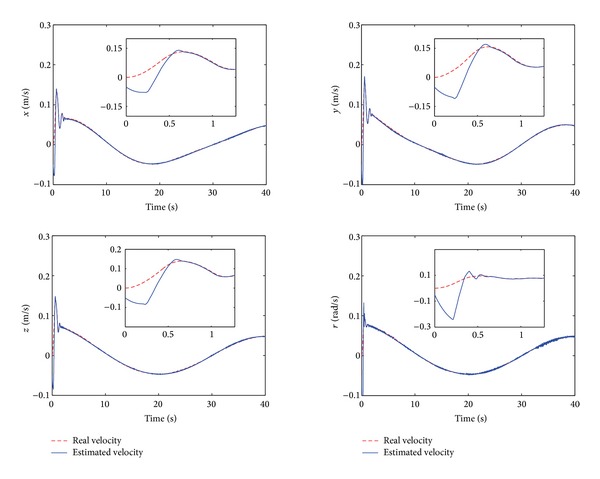
Estimated velocity and real velocity of output feedback IO-NTSMC.

**Figure 6:**
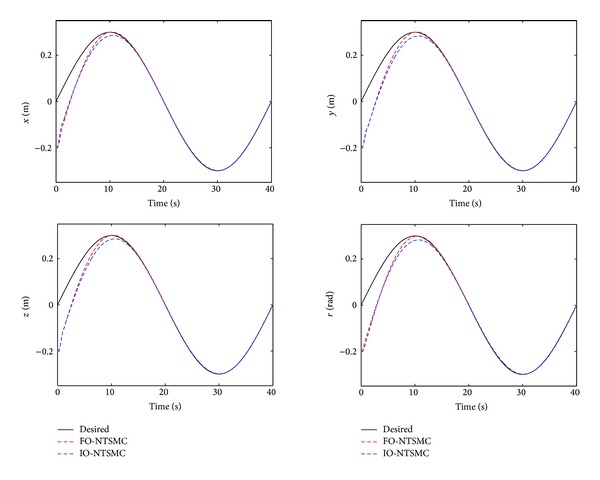
Trajectory tracking performance of output feedback FO-NTSMC and IO-NTSMC.

**Figure 7:**
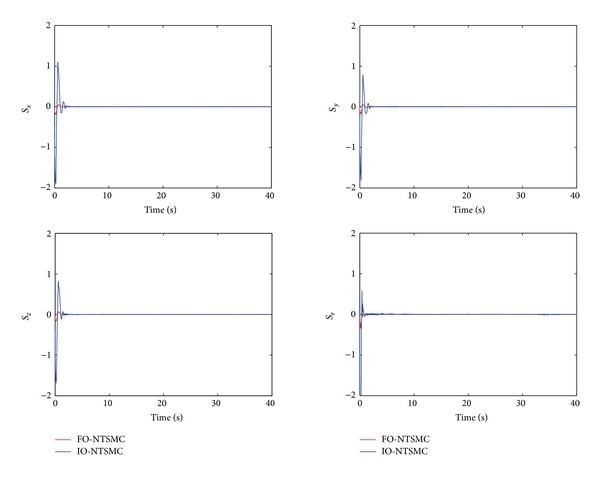
Sliding manifold of output feedback FO-NTSMC and IO-NTSMC.

**Figure 8:**
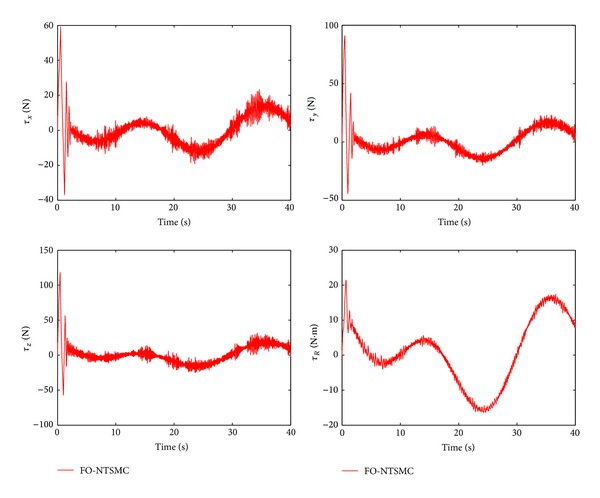
Control inputs of output feedback FO-NTSMC.

**Figure 9:**
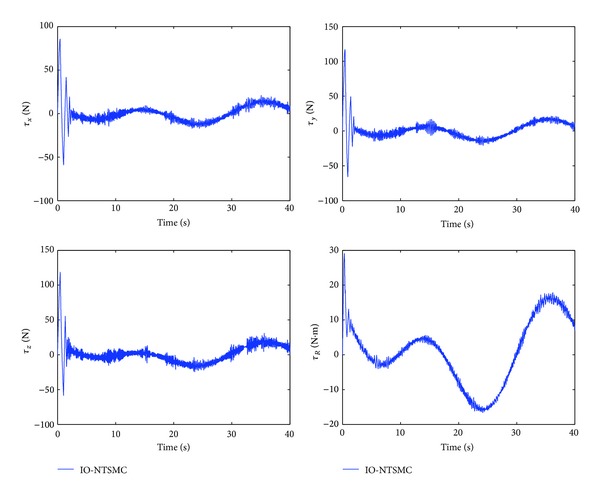
Control inputs of output feedback IO-NTSMC.

The control performance is listed as in Figures 2, 3, 4, 5, 6, 7, 8, and 9. Performance of the proposed equivalent output injection SMO (20) combined with fractional-order/integer-order dynamics is shown in Figures 2–5, respectively. It is clear that the proposed equivalent output SMO can ensure finite-time convergence to the real system states with both fractional-order and integer-order dynamics in the presence of model uncertainties and external disturbances. Figures 6-7 show the trajectory tracking control performance of the output feedback FO-NTSMC and IO-NTSMC. It is clear that the FO-NTSMC can obtain a faster convergence rate and a better dynamic response at the initial stage than the IO-NTSMC, whereas both of them can achieve great robustness against the lumped uncertainties. Furthermore, Figures 8-9 demonstrate that both of the methods have a very serious chattering problem in the control inputs. This problem is mainly caused by the discontinuous terms in the control laws (35) and (52) referred to as *τ*_4_.

**Figure 10:**
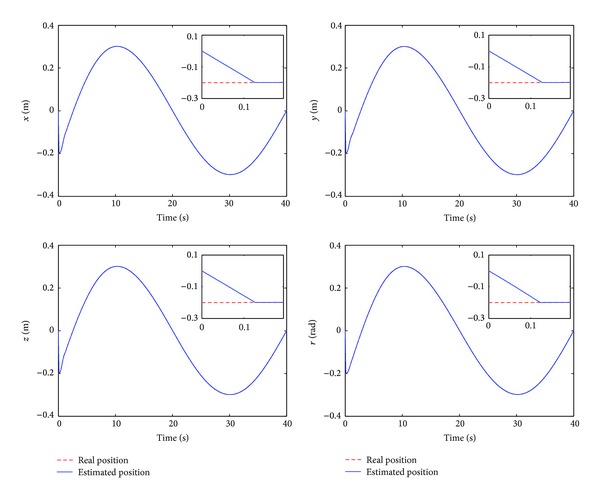
Estimated position and real position of output feedback FO-NTSMC with boundary layer.

**Figure 11:**
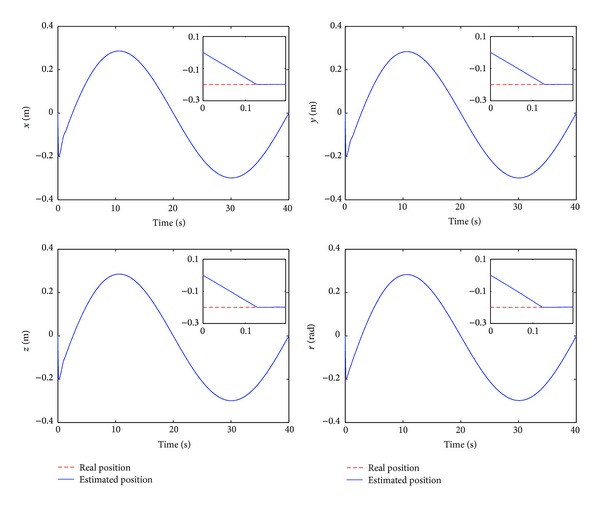
Estimated position and real position of output feedback IO-NTSMC with boundary layer.

**Figure 12:**
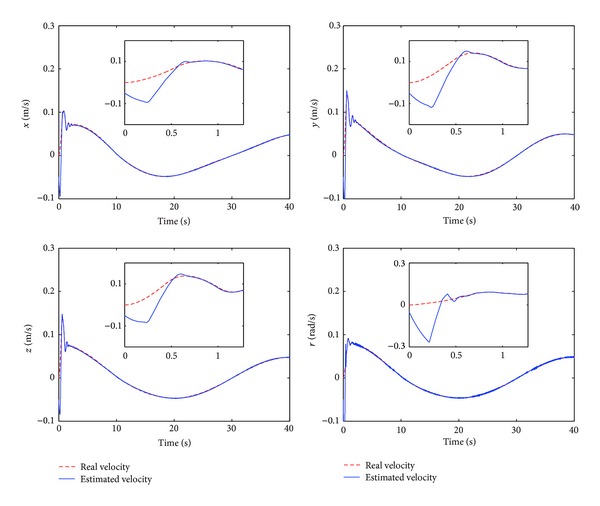
Estimated velocity and real velocity of output feedback FO-NTSMC with boundary layer.

**Figure 13:**
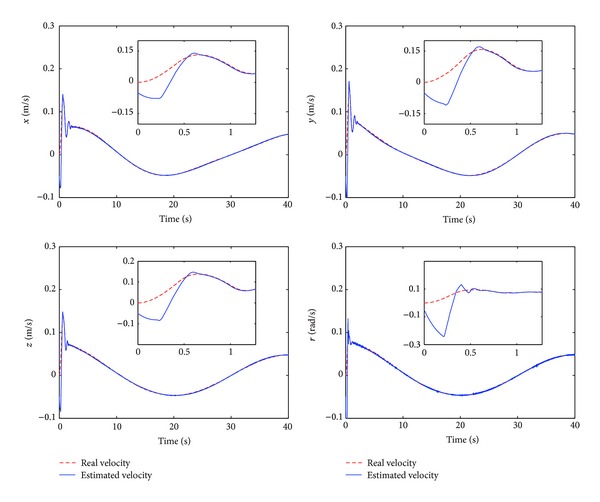
Estimated velocity and real velocity of output feedback IO-NTSMC with boundary layer.

**Figure 14:**
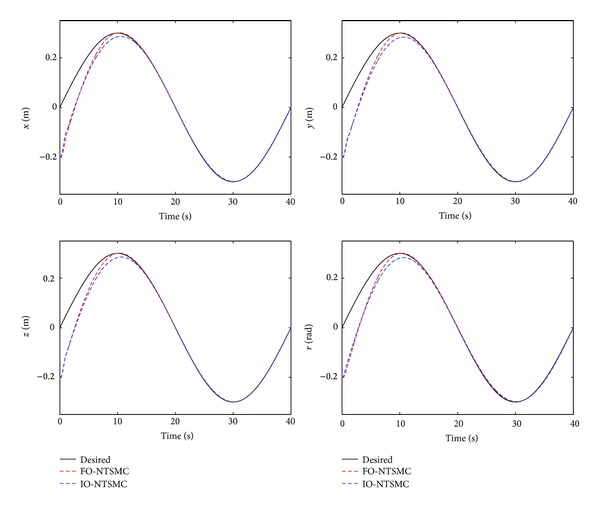
Trajectory tracking performance of output feedback FO-NTSMC and IO-NTSMC with boundary layer.

**Figure 15:**
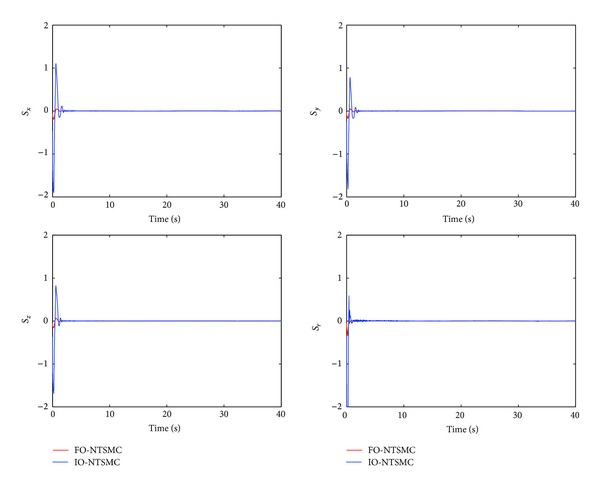
Sliding manifold of output feedback FO-NTSMC and IO-NTSMC with boundary layer.

**Figure 16:**
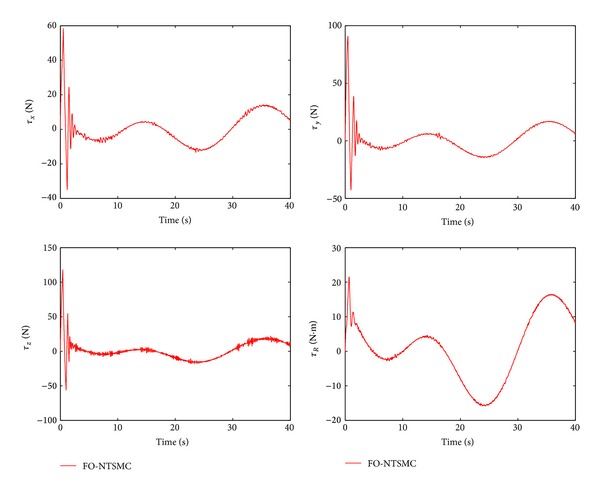
Control inputs of output feedback FO-NTSMC with boundary layer.

**Figure 17:**
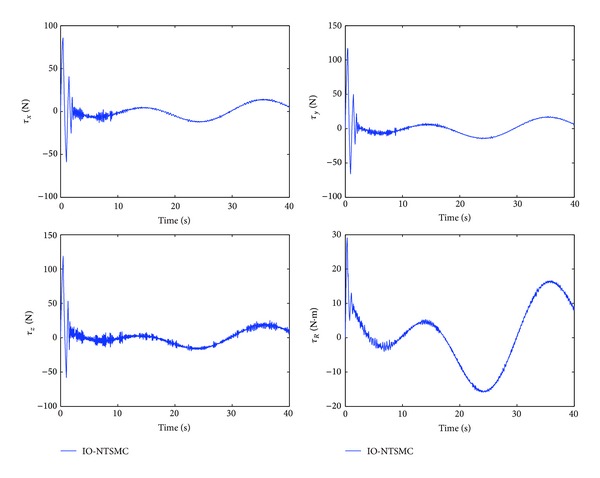
Control inputs of output feedback IO-NTSMC with boundary layer.

To eliminate the chattering phenomenon, the sign functions in *τ*_4_ of the control laws (35) and (52) are replaced by saturation functions with a boundary layer of 0.005. Corresponding simulation results are shown in Figures 10, 11, 12, 13, 14, 15, 16, and 17. It can be clearly observed that the replacement of the sign function does not have an apparent negative effect on the control performance of either method. In addition, the chattering phenomenon is effectively reduced, as shown in Figures 16-17. It is clear that the FO-NTSMC method can still guarantee a faster convergence rate and a better dynamic response than IO-NTSMC with the boundary layer.

## 7. Conclusions

In this study, a novel output feedback FO-NTSMC is designed for classical nonlinear second-order systems of ROVs in light of the equivalent injection SMO and TSMC technology and fractional calculus. The model uncertainties and external disturbances are taken into account throughout the design and analysis procedures. The proposed control scheme can effectively ensure the finite-time stabilization of the closed-loop system using only the plant's output signal. Corresponding stability analysis of the closed-loop system is presented using the fractional-order version of the Lyapunov stability theory. The results of comparative numerical simulation demonstrate the effectiveness and robustness of the proposed control method and its superior performance over that of the output feedback IO-NTSMC.

## Figures and Tables

**Table 1:** Nominal physical parameters of the ROV.

Parameters	Value
*m*/kg	200
*W*/N	2000
*B*/N	2000
*Z*_*B*_/m	−0.108
*I*_*x*_/(kg·m^2^)	25.8
*I*_*y*_/(kg·m^2^)	30.1
*I*_*z*_/(kg·m^2^)	37.8
$X_{\dot{u}}$/kg	−33.6
$Y_{\dot{v}}$/kg	−37
$Z_{\dot{w}}$/kg	−62.9
$N_{\dot{r}}$/(kg·m^2^)	−25
*N*_*r*_/(kg/s)	−170
*Y*_*v*_/(kg/s)	−120
*Z*_*w*_/(kg/s)	−180
*N*_*r*_/(kg/s)	−170
*X*_*u*|*u*|_/(kg/m)	−213
*Y*_*v*|*v*|_/(kg/m)	−270
*Z*_*w*|*w*|_/(kg/m)	−410
*N*_*r*|*r*|_/(kg/m)	−35
